# Ants show a leftward turning bias when exploring unknown nest sites

**DOI:** 10.1098/rsbl.2014.0945

**Published:** 2014-12

**Authors:** Edmund R. Hunt, Thomas O'Shea-Wheller, Gregory F. Albery, Tamsyn H. Bridger, Mike Gumn, Nigel R. Franks

**Affiliations:** 1 School of Biological Sciences, University of Bristol, Life Sciences Building, 24 Tyndall Avenue, Bristol BS8 1TQ, UK; 2 Department of Zoology, University of Oxford, The Tinbergen Building, South Parks Road, Oxford OX1 3PS, UK

**Keywords:** *Temnothorax albipennis*, lateralization, turning bias, thigmotaxis, exploration, mazes

## Abstract

Behavioural lateralization in invertebrates is an important field of study because it may provide insights into the early origins of lateralization seen in a diversity of organisms. Here, we present evidence for a leftward turning bias in *Temnothorax albipennis* ants exploring nest cavities and in branching mazes, where the bias is initially obscured by thigmotaxis (wall-following) behaviour. Forward travel with a consistent turning bias in either direction is an effective nest exploration method, and a simple decision-making heuristic to employ when faced with multiple directional choices. Replication of the same bias at the colony level would also reduce individual predation risk through aggregation effects, and may lead to a faster attainment of a quorum threshold for nest migration. We suggest the turning bias may be the result of an evolutionary interplay between vision, exploration and migration factors, promoted by the ants' eusociality.

## 1. Introduction

Brain lateralization is present in all vertebrate classes and there is now an increasing amount of evidence for sensory and motor asymmetries in the behaviour of invertebrates; this is typically associated with asymmetries in their nervous system [1,2]. Brain regional specialization of tasks is beneficial since it allows lateralized animals to carry out two tasks simultaneously without decreasing their efficiency [3]. For instance, a right eye/left hemisphere bias for identifying prey, and a left eye/right hemisphere bias for predator detection and escape, are reported in fish and lizards, among other vertebrates [4,5].

There is evidence to suggest that population-level behavioural lateralization is more likely to evolve in social than solitary species [6]. Alignment of the direction of behavioural asymmetries is favoured as an evolutionarily stable strategy when asymmetrical individuals must coordinate their behaviours [7]. However, evidence for lateral biases in ants is relatively limited, though their eusociality makes them inviting subjects in which to investigate this hypothesis. The ant *Lasius niger* has been reported to have a population-level preference to keep to the right in densely populated foraging columns on trees, while exposing the left side of their bodies when resting [4]. Population-level asymmetry has also been observed in the red wood ant *Formica aquilonia*, where ants receiving food via trophallaxis use the right antenna to stimulate their donor ant significantly more than the left antenna [8]. Recent research has found that workers of the house-hunting ant *Temnothorax albipennis* seem to rely more on their right eye to recognize landmarks for navigation [9]. This is similar to the finding that bees (*Apis mellifera*) predominantly use their right eye for learning and/or detecting objects [10].

Turning biases in invertebrates have been found using branching maze designs: the common American cockroach (*Periplaneta americana*) has a bias for turning right in a Y-shaped tube, even when an antenna is severed [11], while giant water bugs (*Belostoma flumineum*) show a left-turning bias in underwater T-shaped mazes [12]. Accordingly, we tested the hypothesis that *T. albipennis* shows a turning bias during nest exploration, as a possible result of a lateralized nervous system.

## 2. Material and methods

### (a) Ant colonies

Experiments were carried out in November 2006 on eight colonies collected in September 2006, and in September 2014 on 10 colonies collected in August 2014, from Dorset, UK. Each colony had a queen and was cultured according to established procedures [13].

### (b) Experimental design

**Figure 1.**
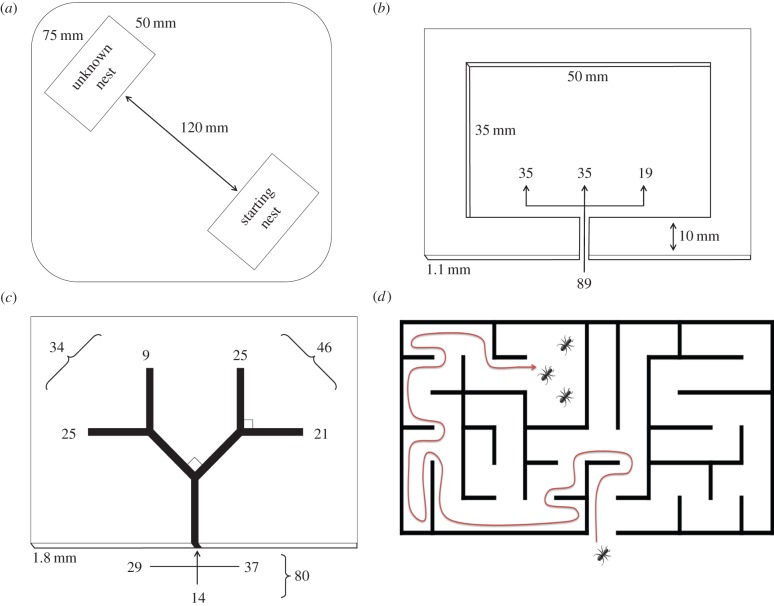
(*a*) The experimental arena layout. (*b*) Ants entering an unfamiliar nest cavity prefer to turn left. (*c*) In a branching cavity there is a left choice bias interacting with a tendency to wall-follow (entry direction numbers left/right or unaligned and choices shown). (*d*) A consistent turning bias favours efficient exploration of an unknown maze-like cavity without getting lost; colony-level turning bias increases the nest-mate encounter rate, which reduces individual predation risk and speeds migration. (Online version in colour.)

In the experiments, ant colonies were placed in a large square Petri dish with Fluon-coated inner walls (230 × 230 × 19 mm). The colony's nest entrance was opposite that of an unknown nest (figure 1*a*). All starting nests were of the same dimensions as the unknown nest in the first experiment (figure 1*a*,*b*), with an entrance width of 1 mm, making them of medium quality according to the ants' preferences [13]. The unknown nest was darker than the starting nest owing to a covering red filter. This rendered it of higher quality and more attractive to scouting ants as *T. albipennis* inhabits dark rock crevices in the wild.

We stimulated exploration of the unknown nest in the first experiment by destroying the starting nest (removing its upper slide). In the second experiment, exploration was encouraged by removing a temporary cardboard cover from the starting nest to increase its light level. After ants had explored and exited the nest, they were removed to a separate holding dish until the end of the experiment to prevent them from participating in a second trial. Clean microscope slides and a replacement perimeter were substituted as a new unknown nest after ant visits to prevent accumulation of pheromones. Used cardboard perimeters were wiped with a damp cloth, while in the second experiment perimeters created from plastic foam were washed with water.

In the first experiment, we observed the initial turning behaviour of scouting ants entering an unknown nest cavity (figure 1*b*). Between 5 and 15 scouting ants were recorded from each of eight colonies, for a total of 89 observations; typical *T. albipennis* colonies have up to a few hundred workers [13]. A directional choice for left or right was determined if an ant remained within a body's width of the wall closest to the entrance, for half the wall's length; otherwise its choice was recorded as ‘other’.

In the second experiment, we used nest cavities with four branches and two decision points, to see whether laterality in choice was sustained (figure 1*c*). The cavities had entrance widths of 2 mm and branch lengths of 10 mm. Ten colonies of 64–166 workers (average 100) were used to collect 8–19 observations per colony, totalling 113 (observation numbers vary owing to differing colony activity levels). Entry direction (via wall-following from the left or right, or unaligned) was not initially noted (*n* = 27 from three colonies) and was occasionally overlooked (*n* = 6), but nevertheless was recorded for the majority of trials (*n* = 80 from seven colonies). Our analysis focuses on this subset of data.

### (c) Statistical analysis

We carried out two-tailed binomial tests on left–right choice data: in the first experiment excluding choices marked ‘other’, and in the second experiment on the totality of second choices, and on choices where thigmotaxis can be excluded (see §3).

A general log-linear analysis was performed on the three factors in the second experiment (entry direction, first choice and second choice), examining the significance of the two- and three-way interaction parameter estimates. A Poisson model was employed. We carried out statistical analyses in SPSS (v. 21).

## 3. Results

In the first experiment, ants were significantly more likely to turn left than right (*p* = 0.0402).

In the second experiment, due to ants' well-established wall-following tendency [14], entry direction information is used to separate the two interacting factors of thigmotaxis (favouring a repeated choice of left–left or right–right) and a bias in favour of leftward choices.

Table 1 and figure 1*c* show the frequencies of first and second choices and entry direction. Table 2 shows the *p*-values associated with the two- and three-way interactions between these factors. An aligned entry direction has a significant interaction with the first choice that an ant makes. This indicates that thigmotaxis is an influential factor in initial nest exploration. However, it does not have a significant interaction with the second choice, although a secondary influence seems likely due to the high numbers of right–right choices (*n* = 17). The relatively small number of unaligned ants (*n* = 14) do not show a statistically significant leftward bias: this may be owing to the small sample size or because more strongly lateralized ants are also those more likely to wall-follow. A leftward turning bias becomes clear by the second choice: first, the sum of all second choices gives 50 left, 30 right (*p* = 0.033) not accounting for higher entry numbers from the right (*n* = 37) than the left (*n* = 29); second, where thigmotaxis can be excluded in the second choice because the ants were observed to (necessarily) detach from the wall at the first choice (left entry, right first choice; right entry, left first choice; unaligned entry), the total is 21 left, 6 right (*p* = 0.006).

**Table 1.** Choice frequency in the second experiment.

entry direction		choice 1		choice 2	
left (L)	29	L	21	L	14
				R	7
		R	8	L	7
				R	1
right (R)	37	L	5	L	5
				R	0
		R	32	L	15
				R	17
unaligned	14	L	8	L	6
				R	2
		R	6	L	3
				R	3
total	80

**Table 2.** Interaction of factors in second experiment, general log-linear analysis.

factor 1	value	factor(s) 2 (and 3)	value	interaction parameter *p*-value
entry direction	unaligned	first choice	left	0.052
		second choice	left	0.884
		first and second choice	left, left	0.398
	left	first choice	left	0.002^a^
		second choice	left	0.071
		first and second choice	left, left	0.056
	right	first choice	right	0.011^a^
		second choice	right	0.26
		first and second choice	right, right	0.056
first choice	left	second choice	left	0.097
	right	second choice	right	0.343

^a^Significance at the 5% level.

## 4. Discussion

The result of the first experiment shows that when entering a relatively unrestrictive cavity, the ants prefer to turn left. The second experiment shows that ants tend to persist in incidental thigmotactic behaviour at the first junction of a narrow, branching nest cavity. However, where thigmotaxis is absent or otherwise diminished in importance before the second choice, a leftward turning bias becomes evident. The bias is strong enough to be significant at the population-level, though a minority of unbiased and right-biased ants could well be present among a majority of left-biased individuals.

Multiple factors may have interacted through natural selection to favour this directional asymmetry in the population-level laterality distribution. Its proximate cause is probably an asymmetrized nervous system indicated by lateralized vision [9] and may tend to operate on a reflexive rather than deliberative level. Progressing into a dark cavity may prompt a switch between behavioural modes and their different brain regions, which could prefer input from particular eyes: from exploring or foraging outside and considered decision-making (right eye) to predator vigilance and readiness for rapid response (left eye). This would seem to favour approaching unfamiliar and potentially dangerous passages on the left and is consistent with observations across multiple ant species of disproportionate appendage severance on the left in interspecific fights, and a greater propensity to turn left when alarmed [4].

A study of ladybirds (*Coccinella septempunctata*) found turning bias at the level of the individual and suggested that this increases both their exploration and foraging efficiency [15]. Considering these aspects of directional choice, an additional ecological factor in favour of a turning bias (left or right) is that nest-seeking colonies of *T. albipennis* need to investigate rock cavities that are typically dark, narrow and partly maze-like, and thus scouts exploring such spaces require a reliable method to ensure that they find their way back to the entrance. One well-known maze-solving algorithm is the ‘wall-follower’ technique. By staying in contact with either the left or right side of the wall of a maze, assuming it is simply connected (walls are contiguous), following the wall will return an explorer to either the same entrance or a different exit; this has been demonstrated concretely in robots [16]. Such a simple left- or right-turn heuristic mitigates the cognitive demand on ants confronted with repeated decision-making. Furthermore, if the turning bias is replicated at the colony level, subsequent scouts entering the same maze would be more likely to encounter their nest-mates (figure 1*d*). This would reduce individual predation risk through aggregation effects, such as Hamilton's ‘selfish herd’ effect whereby animals obtain cover from nearby conspecifics [17]. It may also expedite the attainment of a quorum threshold, speeding the choice of a new nest site for the colony [18]. The collective impact of the individual bias could be further amplified in nature by social pheromone laying [14].

In conclusion, while exploratory turning bias has the potential to enhance the fitness of organisms nesting or foraging in maze-like environments, in solitary species it may have only emerged sporadically at the level of the individual. This is owing to possible costs associated with a lateralized nervous system, such as the related risks of relying more on one eye for predator detection and of exhibiting stereotypical behaviour [4]. The particular benefits of coordinated behaviour associated with the ants' eusocial organization may help to outweigh these costs, favouring the emergence of a population-level turning asymmetry.

## Data accessibility

All data can be found at: http://dx.doi.org/10.5061/dryad.7rq20.
